# Ten-eleven translocation-2-mediated macrophage activation promotes liver regeneration

**DOI:** 10.1186/s12964-023-01407-7

**Published:** 2024-02-02

**Authors:** Yiyuan Chen, Lijun Meng, Nan Xu, Huan Chen, Xuyong Wei, Di Lu, Shuai Wang, Xiao Xu

**Affiliations:** 1 The Fourth School of Clinical Medicine, Zhejiang Chinese Medical University, Hangzhou, 310053 China; 2 Zhejiang University School of Medicine, Hangzhou, 310058 China; 3 Key Laboratory of Integrated Oncology and Intelligent Medicine of Zhejiang Province, Hangzhou, 310006 China; 4 Institute of Organ Transplantation, Zhejiang University, Hangzhou, 310003 China

**Keywords:** Macrophage, Inflammation, Liver regeneration, Tet2, Hepatocellular carcinoma

## Abstract

**Background:**

The remarkable regenerative capacity of the liver enables recovery after radical Hepatocellular carcinoma (HCC) resection. After resection, macrophages secrete interleukin 6 and hepatocyte growth factors to promote liver regeneration. Ten-eleven translocation-2 (Tet2) DNA dioxygenase regulates pro-inflammatory factor secretion in macrophages. In this study, we explored the role of Tet2 in macrophages and its function independent of its enzymatic activity in liver regeneration.

**Methods:**

The model of liver regeneration after 70% partial hepatectomy (PHx) is a classic universal model for studying reparative processes in the liver. Mice were euthanized at 0, 24, and 48 h after PHx. Enzyme-linked immunosorbent assays, quantitative reverse transcription-polymerase chain reaction, western blotting, immunofluorescence analysis, and flow cytometry were performed to explore immune cell infiltration and liver regenerative capability. Molecular dynamics simulations were performed to study the interaction between Tet2 and signal transducer and activator of transcription 1 (Stat1).

**Results:**

Tet2 in macrophages negatively regulated liver regeneration in the partial hepatectomy mice model. Tet2 interacted with Stat1, inhibiting the expression of proinflammatory factors and suppressing liver regeneration. The Tet2 inhibitor attenuated the interaction between Stat1 and Tet2, enhanced Stat1 phosphorylation, and promoted hepatocyte proliferation. The proliferative function of the Tet2 inhibitor relied on macrophages and did not affect hepatocytes directly.

**Conclusion:**

Our findings underscore that Tet2 in macrophages negatively regulates liver regeneration by interacting with Stat1. Targeting Tet2 in macrophages promotes liver regeneration and function after a hepatectomy, presenting a novel target to promote liver regeneration and function.

**Graphical Abstract:**

Tet2 interacts with Stat1 in the cytoplasm and suppresses IFN-γ-induced macrophage activation. Tet2 inhibitor decreases the combination of Stat1 and Tet2, activating the macrophages through the Jak-Stat pathway. The activation of macrophages increases the transcription and translation of the IL-6 and promotes liver regeneration.

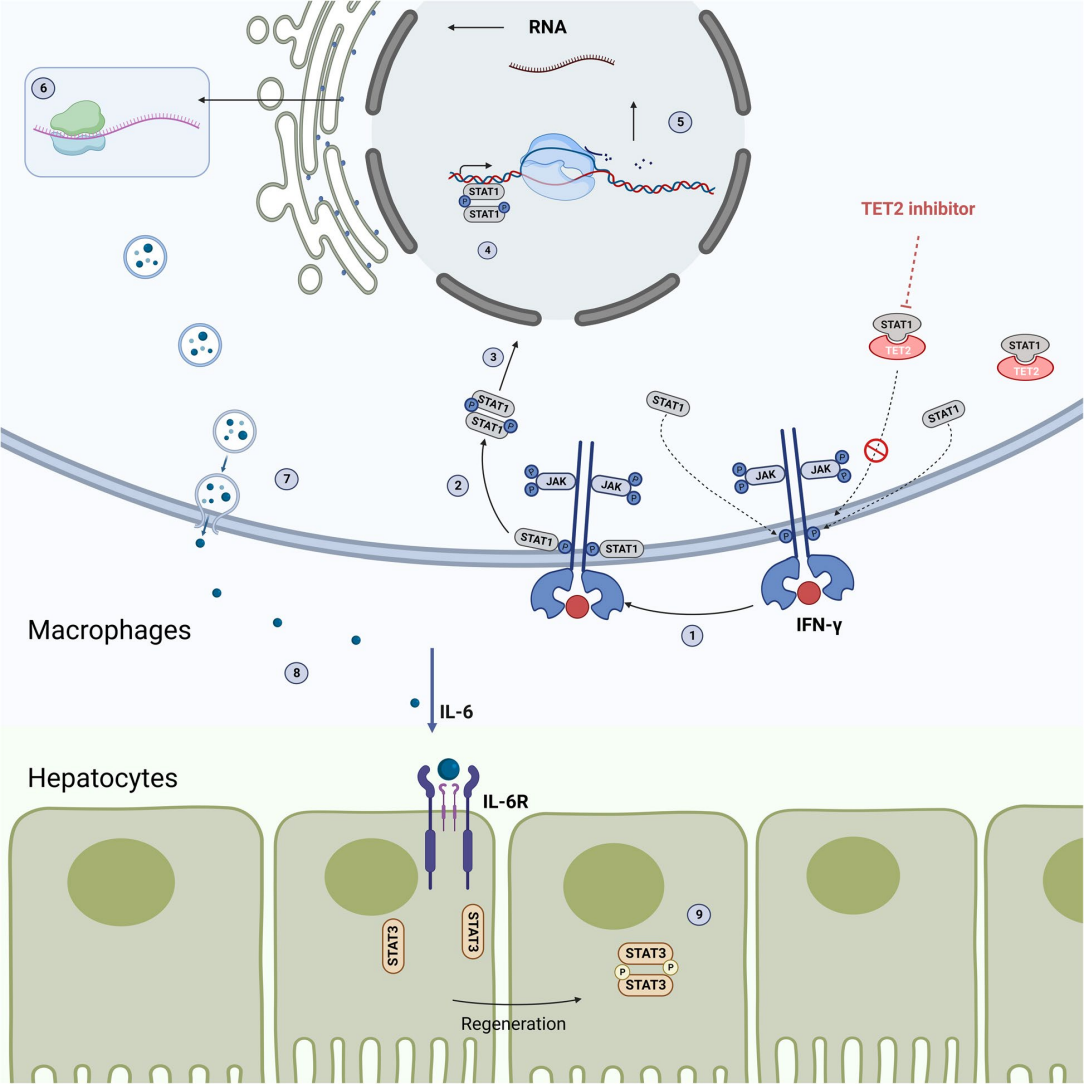

Video Abstract

**Supplementary Information:**

The online version contains supplementary material available at 10.1186/s12964-023-01407-7.

## Background

HCC is the most prevalent primary liver cancer, representing 75–85% of cases [1]. Surgical resection and transplantation are key treatments for early-stage HCC, the 5 years survival rate is achieving 70% [2]. Severe liver injury with fibrosis or steatosis hampers regeneration [3]. Unsuitable liver remnants can cause small-for-size syndrome, leading to poor graft function, longer intensive care unit stays, and recipient death [4]. Therefore, a study on liver regeneration after hepatectomy is of great value.

Liver regeneration is tightly regulated by distinct signaling cascades involving innate immune system components, cytokines, and growth factors [5, 6]. Hepatic immune cells maintain local tissue homeostasis and systemic immunity [7, 8], and various immunotherapies have attempted to modulate the hepatic immune microenvironment to treat liver diseases [9–11].

Liver macrophages comprise ontogenically distinct populations, including resident macrophages (Kupffer cells, KCs) and monocyte-derived macrophages (MoMFs). KCs and MoMFs contribute to interleukin (IL)-6 production and directly promote liver proliferation via the IL-6- signal transducer and activator of transcription 3 (Stat3) pathway [12]. The early activation of KCs secretes IL-6 and hepatocyte growth factor (HGF) [13], accelerating the priming phase and the progression through the G1/S transition after the PHx model [14]. Other products, such as cytokines, chemokines, and reactive nitrogen and oxygen species also influence hepatocyte activity [15]. MoMFs are also beneficial for liver regeneration via transfer into the necrotic foci to phagocytose dead cell debris [16, 17]. When macrophages are depleted, liver regeneration is severely impaired after applying the PHx [18].

As the most canonically epigenetic modification, DNA methylation regulates transcription silencing and genome stability [19], essential for mammalian development [20]. Ten-eleven translocation (Tet) family members are critical enzymes in DNA methylation circulation and correlate with liver disease. Tet2 is a crucial regulator for normal hematopoiesis, especially myelopoiesis. Tet2 binds and recruits histone deacetylase (HDAC1/2) in an enzymatic activity-independent manner to facilitate histone deacetylation and suppress IL-6 and IL-1β expressions during inflammation resolution in innate myeloid cells and macrophages [21, 22].

In this study, we aimed to elucidate the mechanism of Tet2 in macrophages involved in liver regeneration.

## Materials and methods

### Animal experiments and ethics statement

Male C57BL/6 mice (6–8 weeks old) were obtained from the Model Animal Research Center of Nanjing University (Nanjing, China) and randomly assigned to each group of 10 mice. Animal experiments were performed following the Ethics Committee of the Institutional Animal Care and Use Committee (IACUC) (approval number: ZJCLA.ZJCLA-IACUC-20040151). As previously reported by Higgins and Anderson, a modified 70% PHx model was used to investigate and evaluate the complex physiology and pathology of the liver. PHx is a commonly used model in liver regeneration, and its structure is close to clinical liver recession. The model is stable, and the immediate PHx can be used as the starting point of the regeneration process. Mice were intraperitoneally injected with BobCat339 hydrochloride (BC339, 20 mg/kg, S6682, Selleck Chemicals, Houston, TX USA) every other day, one week before the 70% PHx. Mice PHx was performed using inhalation anesthesia with 5% isoflurane during induction and 3% isoflurane for maintenance. The right median (30%), left median (10%) and left lateral liver lobes (30%) were surgically removed while mice were under anesthesia. These lobes were ligated by manipulating each lobe into a preformed loop of silk and tying the loop at the base of each pedicle. The peak of the regeneration phase is 36–48 h in mice. Therefore, we euthanized mice 0, 24, and 48 h after PHx [23–25]. The lobes were weighed, and the liver-to-body weight ratio was calculated to delineate the increasing proportion of liver volume (liver-to-body weight ratio = liver weight/body weight, g/g).

### Depletion of macrophages, NK cells and B cells

Liver macrophages were depleted by intravenously injecting CL (40337ES10, YEASEN, Shanghai, China) at a dose of 50 mg/kg body weight 24 h before and once after PHx. As a control, mice were treated with phosphate-buffered saline (PBS) liposomes (40338ES10, YEASEN). Anti-mouse CD3ε antibody (145-2C11-BP0001-1, Bio X Cell, Lebanon, NH, USA) and anti-mouse NK1.1 antibody (PK136-BP0036, Bio X Cell) were used to deplete T and NK cells by intravenous injection at a dose of 200 µg 24 h before and once after PHx. Mice injected with rat IgG2a isotype control (BP0089, Bio X Cell) were used as controls.

### Cell culture

Primary hepatocytes were isolated from the livers 48 h after PHx and treated with 50 µmol/mL BC339 hydrochloride for 24 h. Mouse primary hepatocytes were isolated from the mice livers by modifying the collagenase method [26]. Livers were perfused with Hank’s balanced salt solution (H4641, Sigma-Aldrich, St. Louis, MO, USA) and washed at 5 mL/min using the portal vein before collagenase (0.025%) treatment. The trypan blue exclusion test was used to assess cell viability (> 60%). Hepatocytes were seeded at a density of 2 × 10^6^ cells/well (in 60-mm Petri dishes for RNA extraction) or 5 × 10^6^ cells/well (in 100-mm Petri dishes for metabolite concentration assays) in Dulbecco’s Modified Eagle Medium (DMEM) with Earle salts (Invitrogen, Waltham, MA, USA), supplemented with 10 g/mL streptomycin and 100 units/mL penicillin. After cell attachment (4 h), the medium was replaced by fresh DMEM for 24 h. The primary hepatocytes were incubated at 37 °C with 5% CO_2_ in a humidified incubator for 24 h, and growth was determined before any intervening measure.

Mouse primary peritoneal macrophages were elicited using a Thioglycolate medium (90,404, Millipore, German). Macrophages were cultured in RPMI 1640 medium containing 10% fetal bovine serum (Gibco, Billings, MT, USA) at 37 °C with 5% CO_2_ in a humidified incubator for 24 h. All culture media was supplemented with 1% penicillin and streptomycin. Macrophages were stimulated with IFN-γ (interferon gamma) for the indicated times (0, 15, 30, 90 min or 0, 3, 6, 9, 12, and 24 h) or with the concentrations (0, 10, 20, 40, 60, 80, and 100 ng/ml).

### Western blotting and immunoprecipitation

Cells were harvested and lysed on ice using cell lysis buffer (#9803, Cell Signaling Technology, CST, Danvers, MA, USA) for 30 min. The protein concentration was measured using a BCA kit (23,227, Thermo Fisher Scientific, Waltham, MA, USA), and protein samples were added to 5 × loading buffer (Beyotime, Jiangsu, China), boiled for 15 min, separated through 10% sodium dodecyl sulfate–polyacrylamide gel, transferred onto polyvinylidene difluoride membranes (Millipore, Burlington, MA, USA), incubated with the indicated primary antibodies at 4 °C overnight and incubated with secondary antibodies (1:2000, CST) for 1 h at room temperature. The signal was detected using an ECL kit (32,106, Thermo Fisher Scientific). The genes were normalized to glyceraldehyde-3-phosphate dehydrogenase (GAPDH) expression levels in each sample. The primary antibodies used were as follows: anti-Tet2 antibody (1:1000, ab124297, Abcam, Cambridge, UK); anti-Stat1 (1:1000, 14,994, CST), anti-pStat1 (Tyr701) (1:1000, 9167, CST), anti-Stat3 (1:1000, 4904, CST), anti-p-Stat3 (1:1000, 9145, CST), anti-Jak1 (1:1000, 3344, CST), anti-Jak2 (1:1000, 3230, CST), and anti-GAPDH (1:2000, ab181602, Abcam). For immunoprecipitation IP (IP) and co-IP assays, cells were lysed with cell lysis buffer (CST) on ice for 30 min and incubated with the indicated antibodies at 4 °C with rotation overnight. The supernatant was then incubated with 10 μL of magnetic beads at 4 °C with rotation for 1 h. The magnetic beads were washed thrice with cold NETN buffer using a magnetic separator (Millipore), followed by elution with 40 μL of protein lysis buffer. 5 × loading buffer (10 µL) was added, and the mixture was boiled for 15 min and subjected to western blotting.

### Quantitative real-time PCR

Total RNA was extracted using Trizol Reagent (Invitrogen) and reverse-transcribed into cDNA using the PrimeScriptTM RT Master Mix kit (Takara, Shiga, Japan). Quantitative real-time PCR (qRT-PCR) was performed using the Q6 real-time PCR system (Applied Biosystems, Waltham, MA USA) with SYBR Green Master Mix (Takara). The data were normalized to GAPDH expression levels. Each group had three biological replicates, and each sample had three technical replicates. The primers for qRT-PCR were designed as follows: Tet2: F: -5’AGAGAAGACAATCGAGAAGTCGG3’-, R: -5’CCTTCCGTACTCCCAAACTCAT3’-; Irf1: F: -5’ATGCCAATCACTCGAATGCG3’-, R: -5’CCTGCTTTGTATCGGCCTGT3’-; Ifit1: F: -5’CCAAGTGCTGCCGTCATTTTC3’-,R: -5’GTGCATCCCCAATGGGTTCT3’-; Cxcl10: F: -5’CCAAGTGCTGCCGTCATTTTC3’-, R: -5’GGCTCGCAGGGATGATTTCAA3’-; Sox9: F: -5’GAGCCGGATCTGAAGAGGGA3’-, R: -5’GCTTGACGTGTGGCTTGTTC3’-; Tert: F: -5’GCACTTTGGTTGCCCAATG3’-, R: -5’GCACGTTTCTCTCGTTGCG3’-; Lgr5:F: -5’CCTACTCGAAGACTTACCCAGT3’-, R: -5’GCATTGGGGTGAATGATAGCA3’-; IL-6: F: -5’ATGGCGTTACTGGATCTGTGC3’-, R: -5’CGCGGAGAAACTGTAGTGTCC3’-.

### Immunohistochemistry and immunofluorescence

Paraffin-embedded liver Sects. (4 µm thick) were used for immunohistochemistry (IHC) experiments. The sections were dewaxed, rehydrated, and quenched with 3% H_2_O_2_, followed by heat-induced epitope retrieval in 10 mM citrate buffer (pH 6) at 95 °C for 20 min. Nonspecific antigens were blocked with 1% BSA (cat: A7906, Sigma-Aldrich). Anti-Ki67 (1:500, ab15580, Abcam) and anti- Hnf4α antibodies (1:500, 3113S, CST) were incubated overnight at 4 °C. Goat-anti-rabbit fluorescein isothiocyanate-labeled IgG or goat-anti-mouse rhodamine IgG (1:200, Proteintech, Rosemont, IL, USA) were incubated at 4 °C for 2 h, followed by 4′,6-diamidino-2-phenylindole (DAPI) staining (Abcam) the cell nucleus. Slides were mounted and visualized using an OLYMPUS microscope.

Primary hepatocytes or macrophages were seeded in a cell culture dish for immunofluorescence experiments. The cells were fixed and permeabilized at 4 °C for 30 min. After incubation with anti-Tet2 (1:100, ab124297, Abcam), anti-Ki67 (1:100, ab15580, Abcam), and anti-Stat1 antibodies (1:100, 9176, CST) at 4 °C overnight, the cells were washed with PBS and stained with goat-anti-rabbit FITC-labeled IgG or goat-anti-mouse rhodamine IgG (1:200, Proteintech) at 4 °C for 2 h, followed by DAPI staining (Abcam). The cells were viewed using a Zeiss Confocal Microscope Imaging System (Carl Zeiss, Jena).

### Enzyme-linked immunosorbent assay

Mice serum liver transaminase alanine aminotransferase (ALT) and aspartate aminotransferase (AST) levels were measured using an ALT and AST assay kit from Nanjing Jiancheng Bioengineering Institute (Jiangsu, China) according to the manufacturer's instructions. IL-6 was measured using an enzyme-linked immunosorbent assay kit from Thermo Fisher Scientific (88–7064).

### Flow cytometry

Livers were isolated from control or PHx mouse livers and digested using a modified liver collagenase perfusion method. Perfused liver cells were filtered through a 70 mm filter (BD Bioscience, Franklin Lakes, NJ, USA). Non-parenchymal cells, including biliary epithelial cells, endothelial cells, hepatic stellate cells, macrophages, neutrophils, and intrahepatic lymphocytes, were purified through low gravity centrifugation (50 × *g* for 1 min, twice) and medium speed gravity centrifugation (500 × *g* for 5 min, once). Live/dead staining was performed using Fixable Viability Stain 780 (565,388, BD Bioscience) and performed in FACS buffer for 30 min at 4℃ at a dilution of 1:1000. For nuclear staining, cells were fixed and permeabilized using BD Cytofix/Cytoperm Plus (555,028, BD Biosciences) and stained according to the manufacturer’s instructions. Antibody staining was performed in FACS buffer for 30 min at 4℃ at a dilution of 1:200. Primary antibodies against CD45 (75–0451-U100, TONBO, Japan), CD11b (85–0112-U100, TONBO), CD3 (50–0032-U100, TONBO), CD8(65–0081-U100, TONBO), CD20 (152,108, Biolegend, San Diego, CA, USA), NK1.1 (17–5941-82, Thermo Fisher Scientific), F4/80 (12–4801-82, 17–4801-80, Thermo Fisher Scientific; 123,137, Biolegend), LY6C (20–5932-U100, TONBO), LY6G (127,633, Biolegend), Tet2 (ab124297, Abcam), and CLEC4F (156,804, Biolegend) were used for flow cytometry analysis and cell sorting. FITC Conjugation Kit (Fast) – Lightning-Link® (ab188285, Abcam) was used to conjugate the first antibody. Cells were sorted using BD Aria III, and the data were generated using FlowJo V10 (https://www.flowjo.com/). The gating strategy is shown in Fig. S2A, and the mean fluorescence intensity (MFI) is shown in Fig. S2B.

### RNA sequencing

We sorted the F4/80^+^CD11b^+^ macrophages at 0, 24, and 48 h after PHx. Total RNA was extracted using Trizol reagent (15,596,018, Thermo Fisher Scientific), and RNA quantity and purity were analyzed using a Bioanalyzer 2100 and RNA 6000 Nano LabChip Kit (5067–1511, Agilent, USA). The sequencing library was constructed with high-quality RNA samples with RIN numbers of > 7.0. Total mRNA was purified and fragmented into short fragments (Magnesium RNA Fragmentation Module (cat: e6150, NEB, Ipswich, MA, USA) at 94℃ for 5–7 min). The RNA was reverse-transcribed to create the cDNA using SuperScript™ II Reverse Transcriptase (1,896,649, Invitrogen) and used to synthesize U-labeled second-stranded DNAs with *E. coli* DNA polymerase I (m0209, NEB), RNase H (m0297, NEB), and dUTP Solution (R0133, Thermo Fisher Scientific). An A-base was added to the blunt ends of each strand, preparing them for ligation to the indexed adapters. Dual-index adapters were ligated to the fragments and AMPureXP beads for size selection. The ligated products were amplified using PCR after heat-labile UDG enzyme (cat: m0280, NEB) treatment of the U-labeled second-stranded DNA. The average insert size for the final cDNA library was 300 ± 50 bp. We then performed the 2 × 150 bp paired-end sequencing (PE150) on an Illumina Novaseq™ 6000 (LC-Bio Technology Co., Ltd., Hangzhou, China). The raw data generated in the study have been deposited at the Gene Expression Omnibus (GEO, GSE233516) and are publicly available.

### Theoretical modeling and molecular dynamics simulation

We focused on human-derived proteins considering the lack of the experimentally resolved crystal structure of mouse Tet2/Stat1 and high sequence compliance between human and mouse Tet2/Stat1. AlphaFold-multimer [27, 28] was applied to predict the complex formed by the two proteins. This complex, however, only contains residues. The organic ligand and metal ions of Tet2 were then added back by aligning the experimentally resolved structure (PDB: 5d9y) with its corresponding predicted structure. The system for all-atom molecular dynamics (MD) simulations was constructed and simulated using the GROMACS package [29], and the trajectories were analyzed using VMD1.9.3 software [30]. The complex was solvated with a 150 mM NaCl electrolyte in a simulation box measuring $9\times 12\times 8 {nm}^{3}$, leading to the entire system having approximately 89,000 atoms. In the simulation, the positions of ${C}_{\alpha }$ atoms belonging to the linker domain and within 0.5 nm of the DNA binding domain, an adjacent linker domain, were constrained.

We applied the CHARMM36 force field [31] for protein and ions and the TIP3P model for water molecules. The forcefield of organic ligand was generated using CGenFF [32]. The simulation time step was 2 fs. The pairwise vdW interaction was calculated using a smooth (0.10–0.12 nm) cutoff. The long-range Coulomb interaction was computed using the particle-mesh Ewald method with a mesh size of 0.1 nm. After minimization and equilibration, production runs were performed in NPT (constant particle number, constant pressure, constant temperature) ensembles. The pressure was maintained at 1 bar using an isotropic Parrinello–Rahman pressostat. The temperature was maintained at 310 K by applying the Nose–Hoover thermostat.

### Statistical analyses

No pre-processing of data was performed. Mice were randomly assigned to different groups for all mice studies. No mice were excluded from the experiments. IHC experiments were performed double-blinded. Each liver sample was imaged and quantified using Image J 1.53t software. All the western blotting, immunofluorescence, IHC, and qPCR data were repeated at least thrice to quantify liver tissues. Data were analyzed using Prism 8.0 software (GraphPad) and presented as the mean values ± SD. An unpaired two-tailed Student’s t-test was used to determine the statistical significance between the two groups. Multiple group data were analyzed using one-way ANOVA. Differences were significant when *p* < 0.05.

## Results

### Macrophages are essential for liver regeneration

**Fig. 1:**
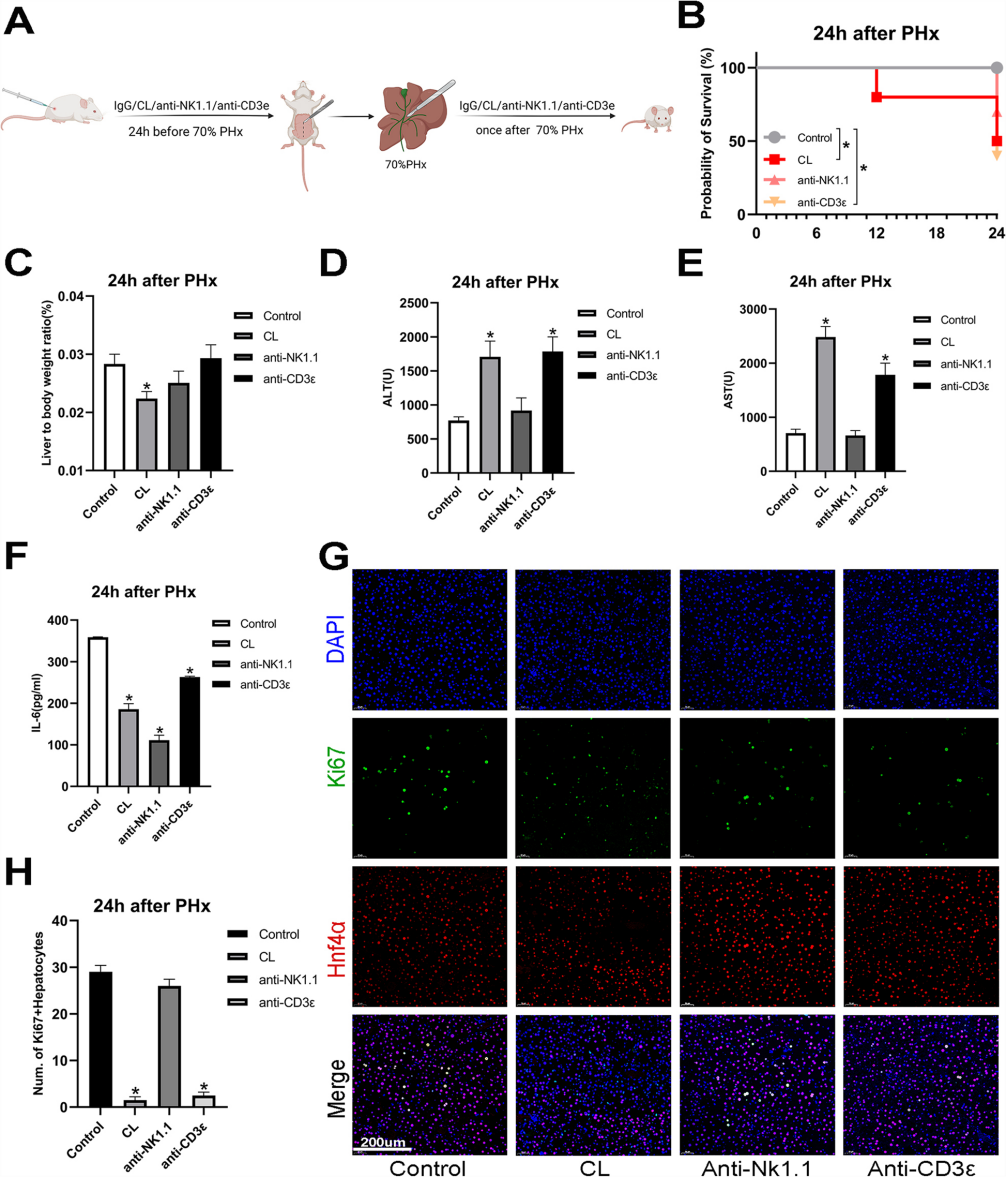
Macrophages play an integral role after partial hepatectomy. Clodronate liposomes (CL), anti-mouse CD3ε antibodies, and anti-mouse NK1.1 antibodies were used to deplete macrophages, T cells, and natural killer (NK) cells in the mouse livers. Mice were anesthetized with isoflurane, and a modified 70% partial hepatectomy (PHx) model was induced. Mice were euthanized 24 h after PHx. **A** Schematic of the macrophages, T cells, and NK cells depletion with PHx. Control: rat IgG2a isotype; CL: clodronate liposomes; anti-CD3ε: anti-mouse CD3ε antibody; anti-NK1.1: anti-mouse NK1.1 antibody. **B** Kaplan–Meier analysis was used to determine the survival of mice 24 h after PHx and immune cell depletion.** C** The liver-to-body weight ratio at 24 h is shown. Liver to body weight ratio = liver weight/body weight (g/g). **D**, **E**, **F** Serum levels of transaminase-alanine aminotransferase (ALT) (**D**), aspartate aminotransferase AST (**E**), and interleukin (IL)-6 (**F**) are shown in the graph. **G**, **H** Co-staining of Ki67^+^ and Hnf4α^+^ (**G**) and the number of Ki67^+^Hnf4α^+^hepatocytes are shown (**H**). (A to B, *n* = 10, C to F, *n* = 6, G to H, *n* = 3. *p < 0.05.)

Since an increased immune response after PHx contributes to liver cell proliferation [33–35], we focused on investigating the roles of immune cells during liver regeneration. We first depleted the macrophages, T cells, and natural killer (NK) cells using clodronate liposomes (CL), anti-mouse CD3ε antibodies, and anti-mouse NK1.1 antibodies, respectively, and constructed a PHx mice model (Fig. 1A). The 24 h survival rate was significantly decreased when macrophages or T cells were depleted (Fig. 1B), consistent with the previously published studies [18, 35]. Furthermore, the liver-to-body weight ratio was severely reduced when macrophages were depleted. Simultaneously, the anti-mouse CD3ε and anti-mouse NK1.1 antibody groups did not show significant differences in the liver-to-body weight ratio compared to the control (Fig. 1C). In addition, the serum ALT and AST levels were markedly increased when macrophages and T cells were depleted, suggesting that liver function is severely impaired in the CL and anti-CD3ε group (Fig. 1D and E).

We further determined the serum IL-6 level, given that IL-6 is critical for hepatocyte cell cycle progression [36]. The serum IL-6 levels were low in all groups, indicating that all immune cell types may contribute to cell cycle progression (Fig. 1F). Moreover, we used the marker of proliferation Ki-67 (Ki67) proliferation marker and hepatocyte nuclear factor 4 alpha (Hnf4α) to count the proliferative hepatocytes in immunofluorescence staining analysis. Immune cell depletion diminished hepatocyte proliferation after PHx (Fig. 1G and H), and the effect of CL was the most significant.

In summary, macrophages might be the most pivotal regulator during liver regeneration, while T cells also contribute to liver regeneration. NK cells did not significantly affect liver regeneration after PHx.

### Macrophage infiltration increases after PHx

**Fig. 2:**
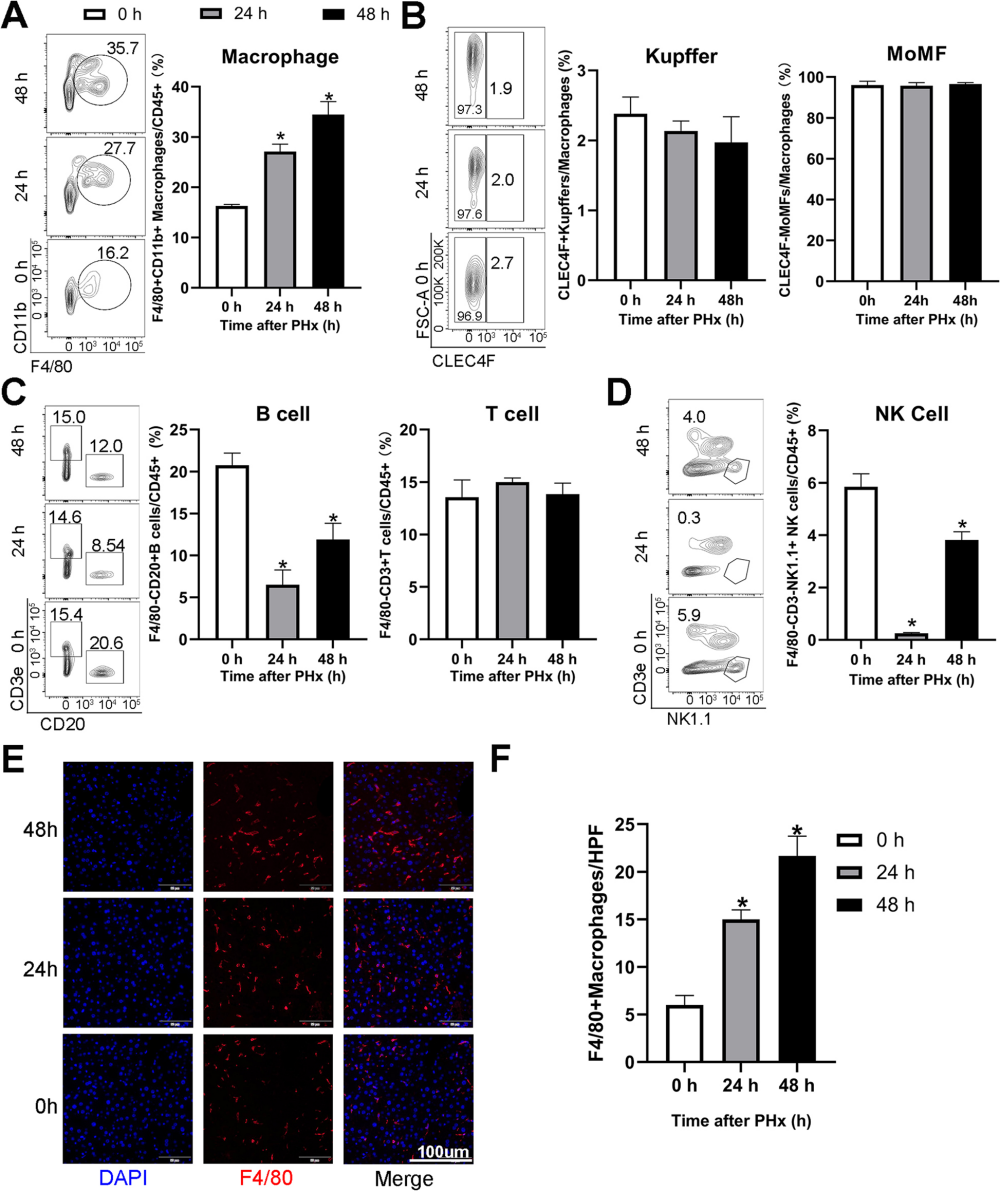
Macrophages increased significantly after PHx. Mice were anesthetized with isoflurane, and a PHx model was constructed. Mice were euthanized, and immune cells were harvested at 0, 24, and 48 h after PHx using a modification of the collagenase perfusion method. Flow cytometry was used to detect the number of each immune cell. The gating strategy is shown in Fig. S2A. **A** Flow cytometry showed the percentage of total macrophages (CD45^+^CD11b^+^F4/80^+^) in total immune cells (CD45^+^). **B** Flow cytometry showed the percentage of Kupffer cells (KCs; CD45^+^CD11b^+^F4/80^+^CLEC4F^+^) and monocyte-derived macrophages (MoMFs; CD45^+^CD11b^+^F4/80^+^CLEC4F^−^) in total macrophages. **C**, **D** Flow cytometry showed the percentage of B (F4/80^−^CD45^+^CD20^+^), T (F4/80^−^CD45^+^ CD3ε^+^), and NK1.1 cells (F4/80^−^CD45^+^NK1.1^+^ CD3ε^−^) in total immune cells. **E**, **F** Immunofluorescent staining of F4/80^+^ macrophages. (A to G, *n* = 6. *p < 0.05.)

We focused on macrophages and their mechanism in regulating liver regeneration. Flow cytometry was used to determine immune cell infiltration, including total liver macrophages (KCs and MoMFs) and B, NK, and T cells after PHx. The gating strategy and MFI are shown in Fig. S2A and B. Flow cytometry analysis showed that the liver total macrophages continuously increased from 0 to 48 h after PHx, whereas the percentage of KCs and MoMFs in total macrophages did not differ significantly after PHx (Fig. 2A and B). In contrast, B cells decreased at 24 h and 48 h (Fig. 2C), possibly a compensatory response for other increasing immune cells. NK cells were almost undetectable at 24 h after PHx but returned to the original level after 48 h (Fig. 2D). Moreover, the total T cells (Fig. 2C) did not show significant variation at either 24 or 48 h. The absolute immune cell numbers per gram liver in 0, 24, and 48 h after PHx are shown in Fig. S2C.

As macrophages had the most significant change, we focused on macrophages and their essential role in liver regeneration. We used immunofluorescence to confirm that macrophages gradually accumulate from 0 to 48 h after PHx (Fig. 2E and F), consistent with the increasing number of macrophages detected using flow cytometry. We further sorted the F4/80^+^CD11b^+^ macrophages at 0, 24, and 48 h after PHx and performed an RNA-seq to clarify the underlying mechanism. Cell division and cycle pathways were significantly enhanced after PHx, explaining the increasing number of macrophages after PHx (Fig. S1A).

### Tet2 expression in macrophages dynamically changes following liver regeneration after PHx

**Fig. 3:**
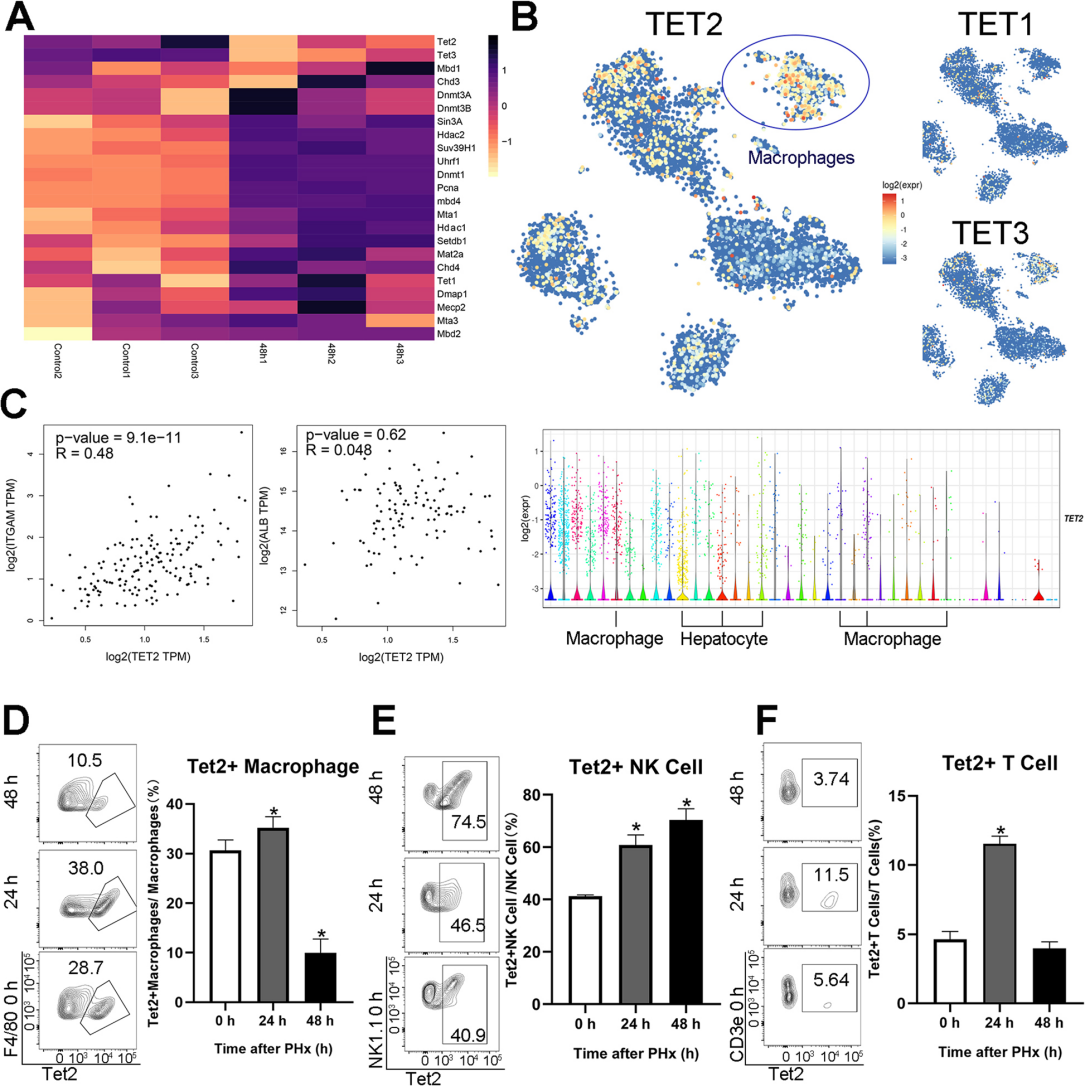
Tet2 negatively regulates liver regeneration after PHx. We analyzed the change in epigenetic factors after PHx using the RNA-seq (GSE158864) and scRNA-Seq databases (GSE124395) published in the Gene Expression Omnibus. Correlation analysis of Tet2 was downloaded from GEPIA. Flow cytometry was used to verify the results. **A** Heatmap of 23 vital epigenetic modification enzymes 0 and 48 h after PHx. **B** Expression t-SNE maps of Tet1, Tet2, and Tet3 in healthy human livers. The color bar indicates log2 normalized expression, and the violin plot of Tet2 expression for all clusters is shown. **C** Correlation analysis showed the Tet2 level with ITGAM (macrophage marker gene) and ALB (hepatocyte marker gene) in normal human liver cells. **D**, **E**, **F** Flow cytometry showing the percentage of Tet2^+^ macrophages (Tet2^+^F4/80^−^CD45^+^CD11b^+^F4/80^+^), Tet2^+^NK cells (Tet2^+^F4/80^−^CD45^+^NK1.1^+^CD3ε^−^), and Tet2^+^T cells (Tet2^+^ F4/80^−^CD45^+^ CD3ε.^+^) in each immune cell type (D to F, *n* = 6. *p < 0.05)

We re-analyzed the published RNA-seq database with PHx (GSE158864) to investigate the change of epigenetic factors during liver regeneration. Tet2 markedly decreases at 48 h after PHx, the peak proliferation time during liver regeneration (Fig. 3A). We suspected Tet2 may act as a negative immune response regulator in liver regeneration after PHx. Therefore, we analyzed the TCGA database to delineate the Tet2 expression in the liver. Tet2 was primarily located in macrophages (Fig. 3B). Furthermore, correlation analysis using the TCGA database indicated that Tet2 expression was positively related to *ITGAM* (integrin alpha M, a macrophage marker gene) but not *ALB* (albumin, a hepatocytes marker gene) in normal liver cells (Fig. 3C), indicating that Tet2 was likely distributed in macrophages rather than in hepatocytes. In addition, the relative mRNA level of Tet2 in whole liver tissue decreased at 24 h and dramatically declined at 48 h (Fig. S1B). Finally, Tet2^+^ macrophages significantly reduced at 48 h; however, total macrophage expression increased (Figs. 2A and 3D). The Tet2^+^T and Tet2^+^NK cells also differed significantly after PHx (Fig. 3E and F). Our results suggest that Tet2 in macrophages dramatically decreased in 48 h and may act as a negative regulator in liver regeneration after PHx.

### Tet2 interacts with Stat1 in the cytoplasm and suppresses IFN-γ-induced macrophage activation

**Fig. 4:**
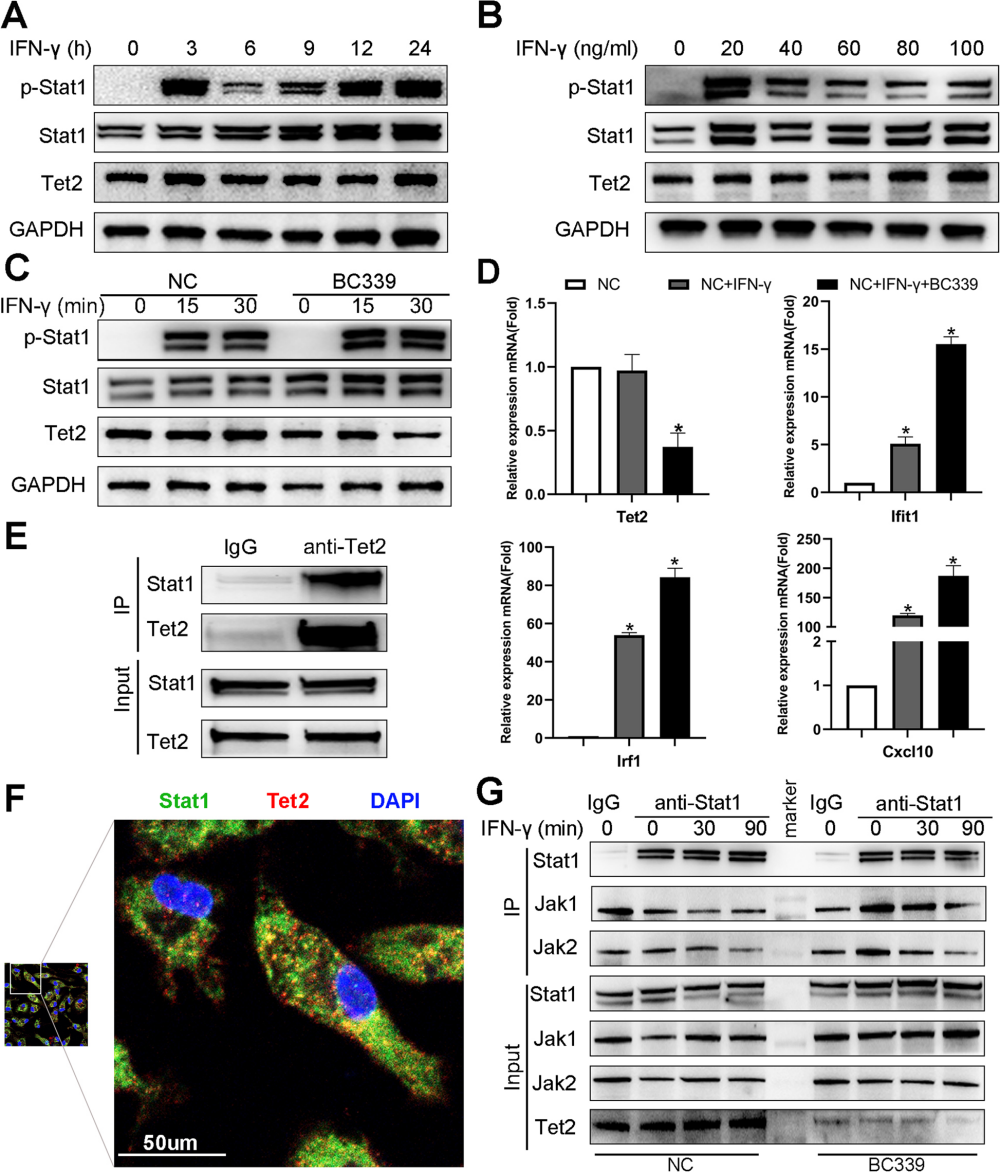
Tet2 interacts with Stat1 in the cytoplasm of macrophages and suppresses Stat1 phosphorylation during IFN-γ signaling activation. **A**, **B** Macrophages were stimulated with 10 ng/mL IFN-γ for the indicated times (**A**) or with the indicated IFN-γ concentrations for 24 h (**B**). Tet2, Stat1, p-Stat1 (Tyr701), and the GAPDH protein level were detected using western blotting. The protein was normalized to GAPDH expression levels. Tet2: ten-eleven translocation- 2, Stat1: signal transducer and activator of transcription 1, p-Stat1: phosphor-Stat1, GAPDH: glyceraldehyde-3-phosphate dehydrogenase. **A** Macrophages were treated with Tet2 inhibitor-BC339 or DMSO as a control for 24 h and stimulated with 10 ng/mL IFN-γ for the indicated time. Tet2, Stat1, p-Stat1 (Tyr701), and GAPDH protein levels were detected using western blotting. The protein was normalized to GAPDH expression levels in each sample. **B** Macrophages were treated with Tet2 inhibitor-BC339 or DMSO as a control for 24 h and stimulated with 10 ng/mL IFN-γ for 12 h. The mRNA expression levels of Tet2, Irf1, Ifit1, and Cxcl10 were examined through qPCR analysis. The genes were normalized to GAPDH mRNA levels in each sample. Irf1: interferon regulatory factor 1, Ifit1: interferon-induced protein with tetratricopeptide repeats 1, Cxcl10: C-X-C motif chemokine ligand 10. **C** Co-immunoprecipitation (co-IP) assays were performed using an anti-Tet2 antibody and IgG as a control. Tet2 and Stat1 protein levels were detected using western blotting. **D** Macrophages were fixed and incubated with an anti-Tet2 antibody (red), anti-Stat1 antibody (green), and DAPI (blue) and visualized using confocal microscopy. **E** Macrophages were treated with Tet2 inhibitor-BC339 or DMSO as a control for 24 h and stimulated with 10 ng/mL IFN-γ for the indicated time. Co-IP assays were performed using an anti-Stat1 antibody and IgG as control. Tet2, Stat1, Jak1, and Jak2 protein levels were detected using western blotting. (Jak1: Janus kinase 1, Jak2: Janus kinase 2, A to G, *n* = 3 *p < 0.05)

Because IFN-γ was initially identified as a ‘macrophage-activating factor,’ and macrophages are a primary physiological target for IFN-γ action [37], we further investigated the role of Tet2 in macrophages stimulated with IFN-γ. Unexpectedly, IFN-γ stimulation had no significant effect on Tet2 protein expression in a time- or dose-dependent manner (Fig. 4A and B). However, Stat1 and phospho-Stat1 (p-Stat1, Tyr701) expression was significantly increased after Tet2 inhibitor BC339 treatment **(**Fig. 4C). Furthermore, inhibiting Tet2 promoted IFN-γ signaling activation and its downstream gene expressions, including interferon regulatory factor 1 (Irf1), IFN-induced protein with tetratricopeptide repeats 1 (Ifit1), and C-X-C motif chemokine ligand 10 (Cxcl10) (Fig. 4D).

We performed co-immunoprecipitation (IP) analysis to investigate the molecular mechanism of Tet2 regulating the IFN-γ pathway and identified Stat1 as one of the primary binding partners of Tet2 (Fig. 4E). Tet2 can form a complex with Stat1 in the cytoplasm of primary macrophages **(**Fig. 4F), indicating that Tet2 which regulates macrophage responses to IFN-γ-induced inflammatory responses might be independent of its enzymatic activity as an epigenetic regulator in the nucleus. Moreover, inhibiting Tet2 increased the association between Janus kinase 1 (Jak1) or Jak2 and Stat1 (Fig. 4G). These results indicate that Tet2 suppresses IFN-γ-induced Stat1 phosphorylation and macrophage activation by hindering Jak1/Jak2 binding to Stat1 (Graphical abstract).

### Molecular dynamics simulations provide insights into the interaction mechanism between Tet2 and Stat1

We performed MD simulations to study the interaction mechanism between Tet2 and Stat1. MD simulations can detect atomic scale and dynamics information of protein–protein interactions, which is challenging to obtain via experiments [38].

**Fig. 5:**
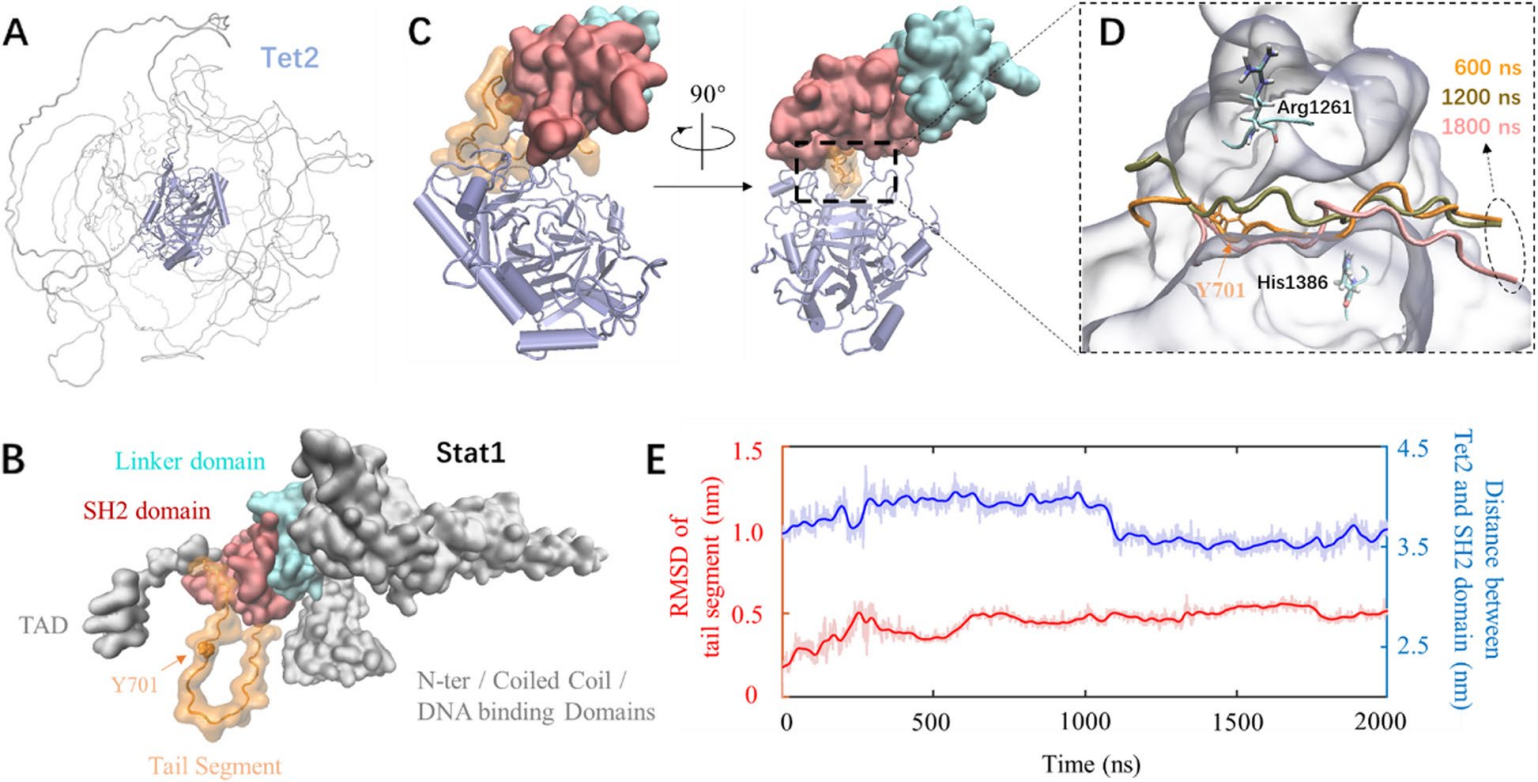
Theoretical modeling and molecular dynamics simulation of the interaction between Tet2 and Stat1.** A** 3D structure of Tet2 predicted using AlphaFold2. The central structured region is in cartoon representation and rendered in light blue. The intrinsic disorder region is in tube representation and colored in gray.** B** 3D structure of Stat1 predicted using AlphaFold2. The tail segment has a transparent surface and tube representation; the remaining domains are opaque.** C** The complex structure (formed by the structured region of Tet2 and the SH2/linker domains plus the tail segment of Stat1) was predicted using AlphaFold-multimer. The region/domains are rendered using the same color code as in (**A**) and (**B**).** D** Enlargement of the interaction interface between Stat1 and Tet2. Tet2 is in light blue transparent surface representation. Three tubes show conformations of a part of the tail segment of Stat1 embedded in the pocket at three simulation times. Two residues (Arg1261/His1386) in stick representation are involved in the interaction between Tet2 and its inhibitor BC339.** E** Red curve: Root-mean-square deviation (RMSD) of the tail segment embedded in the active pocket as shown in (**D**); Blue curve: distance between the center of mass of the structured region of Tet2 and that of the SH2 domain of Stat1. The light (deep) color indicates the original (smoothed) data

The entire Tet2 structure (Fig. 5A) predicted by AlphaFold2 [39] can be roughly divided into two regions: a structured region (blue) containing an active pocket and an intrinsic disorder region (gray). Our experiment, combined with a recent publication [40], has indicated that the BC339 inhibitor can attenuate the interaction between Tet2 and Stat1 by binding to the active pocket of Tet2. Therefore, for Tet2, we chose its structured region for complex modeling and MD simulations. Figure 5B depicts the entire structure of Stat1 predicted by AlphaFold2. During Stat1 activation, it is first recruited to the cytokine receptor binding site via the SH2 domain, and then its Y701 residue located in the tail segment becomes phosphorylated. This phosphorylation further induces Stat1 dimerization and conformational change necessary for translocation into the nucleus and gene transcription regulation [41, 42]. Therefore, for Stat1, the SH2 domain and tail segment were included in the following modeling/simulations. The adjacent SH2 domain (i.e., linker domain) was also included.

AlphaFold-multimer [27, 28] was used to predict complexes formed by the structured region of Tet2 and the linker/SH2 domains plus the tail segment of Stat1. Each of totally five AlphaFold2 models predicted a complex, respectively. In four of the five generated complexes, the structured region of Tet2 was predicted to bind to the linker domain in a conformation that inhibits the linker domain from connecting to its adjacent domain (i.e., DNA binding domain). Thus, only one predicted complex is sterically reasonable, wherein the tail segment of Stat1 is partially embedded in the active pocket (Fig. 5C). Based on this complex, a system for MD simulation was constructed, and an extensive simulation of two microseconds in length was performed.

In addition, Fig. 5D shows the conformational change of a part of the tail segment (in tube representation) directly interacting with Tet2 (in transparent surface representation). The segment is consistently embedded in the pocket, implying a relatively stable binding between the two proteins. As mentioned above, in a recent publication [40], the Tet2 inhibitor molecule BC339 binds to a region inside the active pocket and between Arg1261 and His1386 (see Fig. 3(d) therein). Therefore, the binding location of BC339 overlaps with that of the tail segment of Stat, which also strongly correlates with our result that BC339 can attenuate the interaction between Tet2 and Stat1 (see discussion below). The red curve in Fig. 5E depicts root-mean-square deviation (RMSD) of the embedded tail segment (as shown in Fig. 5D) throughout the simulation trajectory. The fluctuation gradually declined, implying a stable contact at the interface between the two proteins. The distance between the center of mass of the structured region and that of the SH2 domain is also presented using a blue curve. The distance fluctuated around the initial value, again indicating stable.

### Tet2 inhibitor activates macrophages and promotes liver regeneration

**Fig. 6:**
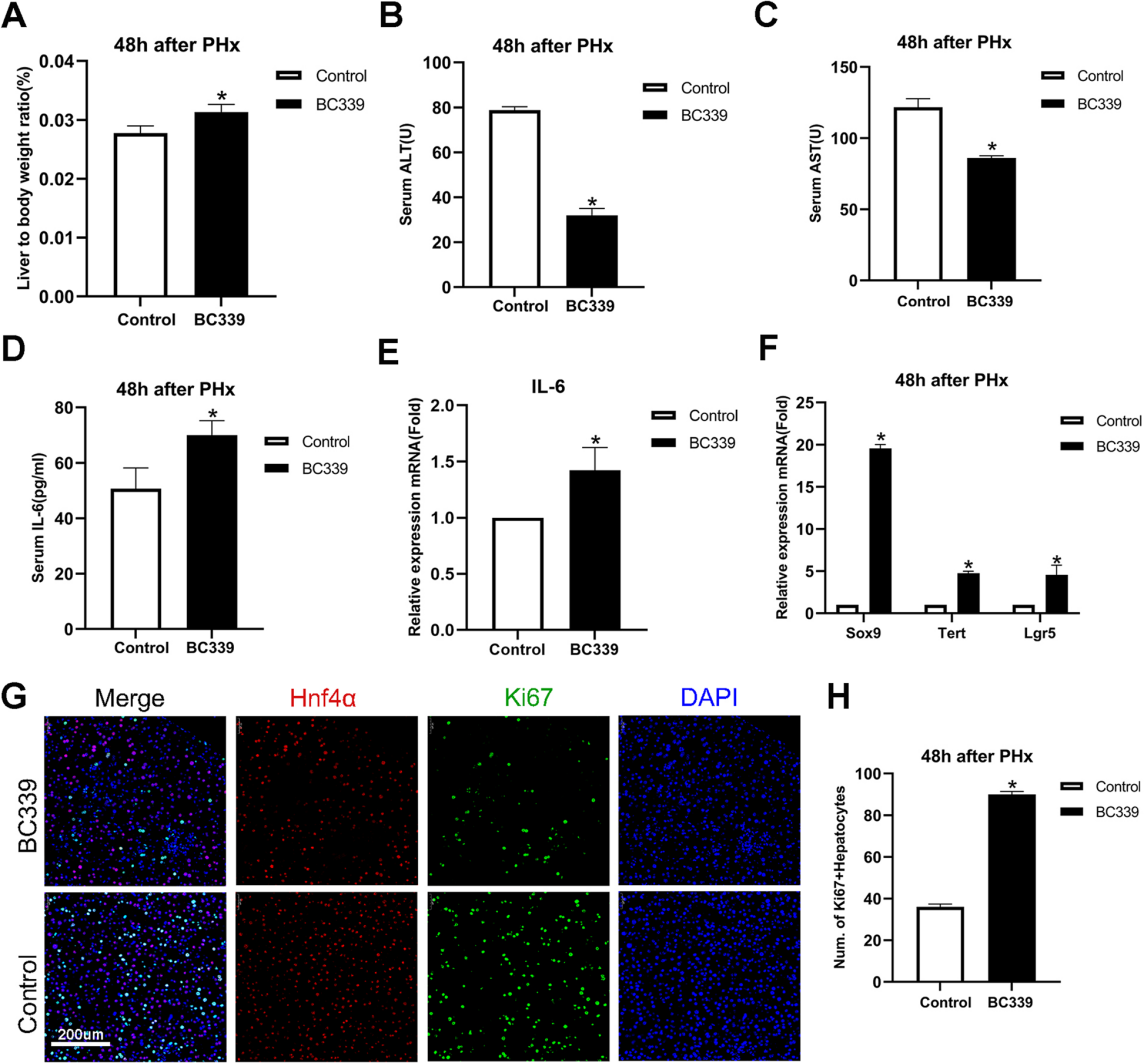
Tet2 inhibits macrophages secreting IL-6 and decreases liver regeneration Mice were intraperitoneally injected with Bobcat339 (BC339; 20 mg/kg) every other day for 1 week, and PHx was performed. Mice were euthanized at 48 h after PHx. Whole liver tissue was harvested 48 h after PHx. **A** Liver-to-body weight ratio at 48 h. Liver to body weight ratio = liver weight/body weight (g/g). **B**, **C**, **D** Serum levels of ALT (**B**), AST (**C**), and IL-6 (**D**) were measured 48 h after PHx. **E**, **F** The mRNA expression levels of IL-6 (**E**), Sox9, Tert, and Lgr5 (**F**) were examined using qPCR analysis (The genes were normalized to GAPDH mRNA levels in each sample. Sox9: SRY-box transcription factor 9, Tert: telomerase reverse transcriptase, Lgr5: leucine-rich repeat-containing G protein-coupled receptor 5). **G**, **H** Co-staining of Ki67 + and Hnf4α + (**G**) and the ratios of Ki67 + Hnf4α + hepatocytes (**H**). Ki67: proliferation marker (Ki-67), Hnf4α: hepatocyte nuclear factor 4 alpha. A to D, *n* = 6, E to H, *n* = 3, *p < 0.05.)

Next, we tentatively explored the role of Tet2 chemical inhibitors (BC339) on liver regeneration. We administered BC339 every other day for 1 week before PHx to evaluate the function of Tet2 in vivo. Because mouse liver regeneration peaks at 36–48 h after PHx, we euthanized mice 48 h after PHx. The 48 h survival ratio was improved by BC339 (Fig. S1C). The liver-to-body weight ratio was significantly increased after BC339 treatment (Fig. 6A). The ALT and AST serum levels decreased in the BC339 group, indicating that BC339 improved liver function in vivo (Fig. 6B and C). Furthermore, IL-6 mRNA expression in the liver tissue and the serum IL-6 significantly increased in the BC339 group after PHx (Fig. 6D and E). In addition, mRNA levels of liver stem cell markers, such as SRY-box transcription factor 9 (Sox9), telomerase reverse transcriptase (Tert), and leucine-rich repeat-containing G protein-coupled receptor 5 (Lgr5), increased in the BC339 group (Fig. 6F), suggesting that BC399 may accelerate the hepatocytes de-differentiated into liver progenitor cells (LPCs) earlier to support liver regeneration [43]. Co-staining Ki67 and Hnf4α using immunofluorescence also confirmed that liver regeneration was strengthened after BC339 treatment (Fig. 6G and H). Therefore, the Tet2 inhibitor BC339 promotes liver regeneration and maintains liver function after PHx.

### Tet2 chemical inhibitors regulating liver regeneration rely on macrophages

**Fig. 7:**
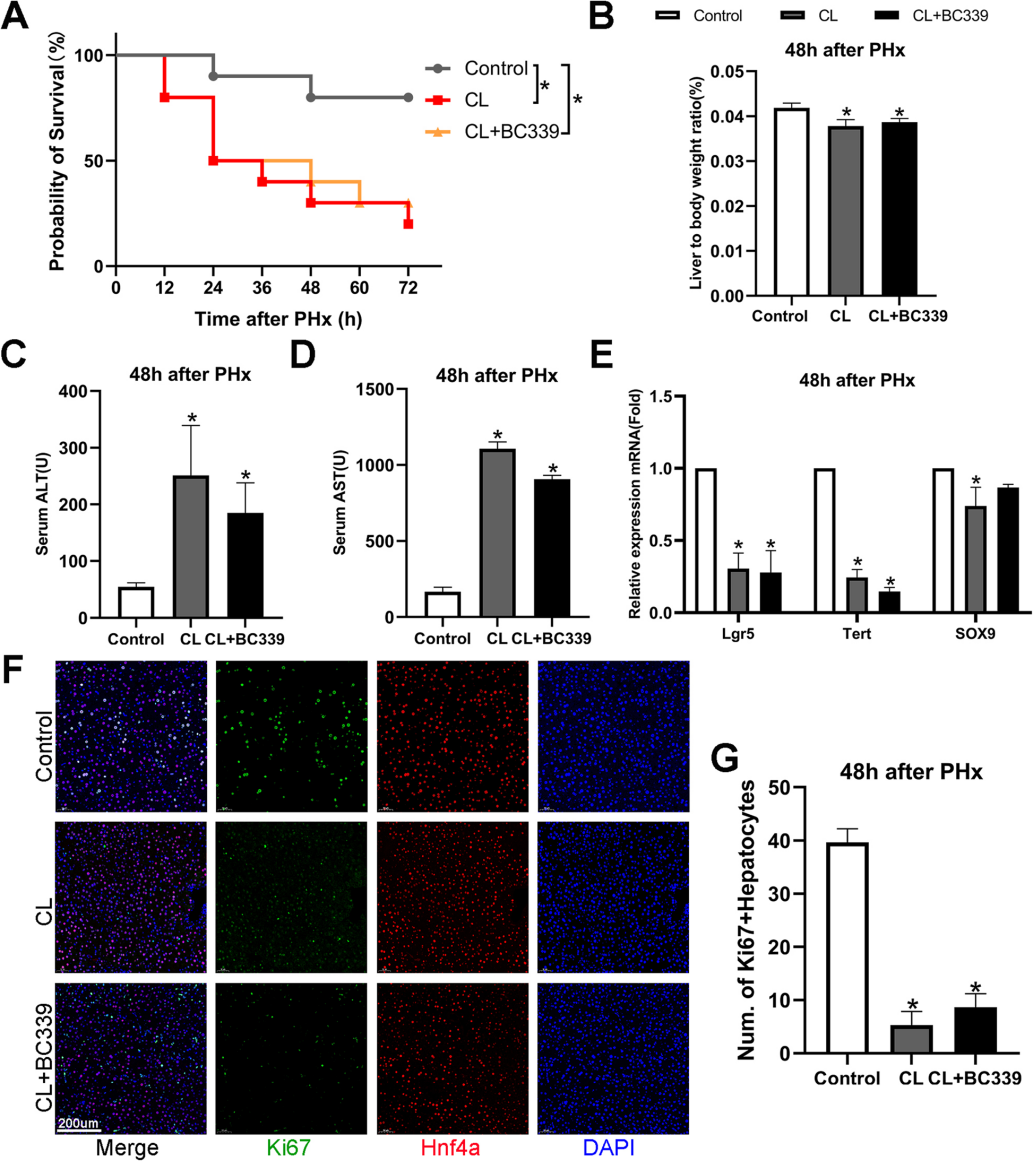
Tet2 chemical inhibitors promote liver regeneration dependent on macrophages. Mice were intraperitoneally injected with BC339 (20 mg/kg) every other day for 1 week. CL was used to deplete macrophages before and after PHx. We performed a modified 70% partial hepatectomy (PHx) model. Whole liver tissue was harvested 48 h after PHx. **A** Kaplan–Meier analysis was used to determine the survival rate of mice after PHx with the depletion of macrophages. We observed the survival rate of mice every 12 h and up until 72 h.** B** Mice were euthanized at 48 h after PHx. The liver-to-body weight ratio. Liver to body weight ratio = liver weight/body weight (g/g). **C**, **D** ALT (**C**) and AST (**D**) serum levels were measured 48 h after PHx. **E** The Tert, Lgr5, and Sox9 mRNA expression levels were examined using qPCR analysis. The gene was normalized to GAPDH mRNA levels in each sample. **F**, **G** Co-staining of Ki67 + and Hnf4α + (**F**) and the ratios of Ki67 + Hnf4α + hepatocytes are shown (**G**). (A, *n* = 10, B to D *n* = 6, E to G, *n* = 3, *p < 0.05.)

Since Tet2 was highly expressed in the macrophages, we explored whether the promotive effect of Tet2 on liver regeneration depends on macrophages. We used CL to remove liver macrophages before and after PHx and treated the mice with BC339 one week in advance. The survival rate of the mice after PHx was observed. BC339 treatment did not promote liver regeneration or improve the survival ratio after exhausting macrophages (Fig. 7A). The liver-to-body weight ratio (Fig. 7B) and the serum ALT and AST levels (Fig. 7C and D) also showed that liver regeneration and function were severely impaired when macrophages were depleted. Immunofluorescence of Ki67^+^Hnf4α^+^ hepatocytes in liver tissue indicated that BC339 could not promote hepatocyte proliferation after CL treatment (Fig. 7F and G). Interestingly, the Sox9, Lgr5, and Tert mRNA levels significantly decreased, suggesting that the Tet2 inhibitor did not promote hepatocytes de-differentiation into LPCs in the early regeneration phase after macrophage depletion (Fig. 7E). Because BC339 promotes IL-6 secretion from macrophages (Fig. 6), we further investigated whether BC339 directly promoted hepatocyte proliferation. We isolated primary hepatocytes from mice after PHx and stimulated them with IL-6. Stat3 and p-Stat3 were significantly activated in primary hepatocytes stimulated with IL-6 at different concentrations (Fig. S2D). However, BC339 did not considerably change Stat3 and p-Stat3 in hepatocytes (Fig. S2E).

Our results illustrate that BC339 contributes to liver regeneration and promotes hepatocyte reprogramming into LPCs through macrophages. In addition, the function of Tet2 relied on macrophages and had no direct effect on hepatocytes.

## Discussion

Our study focuses on liver regeneration after PHx, which is essential for patients with liver diseases like HCC. HCC is the most common liver cancer, often related to hepatitis B in China [44]. Surgical options like resection and transplantation offer hope, but inadequate future liver remnant can lead to complications.

Hepatocyte reprogramming is associated with dynamic immune responses [45, 46]. Aberrant DNA methylation is linked to various pathological diseases, such as cancer [47], obesity [48], and inflammatory autoimmune disorders [49]. Epigenetic regulation also affects liver regeneration after PHx [50–52], and mice transplanted with Tet2-deficient hematopoietic cells display heightened macrophage cytokines [51]. Our study reveals that Tet2 in macrophages negatively regulates liver regeneration. Furthermore, Tet2 in macrophages inhibits Stat1 phosphorylation by interacting with Stat1 in the cytoplasm independent of methylase function. Sterile inflammation is common in injuries and can stimulate regeneration [53], and our findings confirmed that pro-inflammatory signals can directly induce liver regeneration. Moreover, the adoptive transfer of Tet2-deficient B cells inhibits IL-10 expression, suppresses HCC progression, and improves anti-PD-1 (programmed cell death protein 1) therapy for HCC [50]. Therefore, Tet2 inhibition offers anti-HCC effects and enhances liver regeneration after PHx, which could be a novel target for liver regeneration in patients with surgical indications. Itaconic acid (ITA), an intermediate product of the tricarboxylic acid cycle, is reportedly a potent inhibitor of the Tet-family DNA dioxygenases, suggesting that ITA could be a potential approach to promote liver regeneration after PHx [54]. Serum ITA level can be used as a latent predictor to assess the ability of liver regeneration after PHx in clinical application.

Our study also confirmed the published results that T cells contribute to liver regeneration during PHx [35]. T cells synergize with the cellular immune system to promote hepatocyte regeneration. T cell receptor (TCR)-β chain-deficient mice had a reduced liver regeneration capacity, characterized by impaired IL-6 secretion by macrophages and enhanced NK cell activation during PHx. Mice lacking nearly all T cell types display 75% mortality after PHx, whereas those lacking only γδ T cells exhibit impaired hepatocyte proliferation during liver regeneration [55]. TCR-β deficiency also activates the NK and NK T cells in the liver, reportedly reducing liver regeneration during PHx [56]. Our results only focused on NK cells, and NK cell depletion did not significantly affect liver regeneration. In summary, the synergic action between immune cells promotes liver regeneration, and the T cell activation may promote liver regeneration through macrophages. Hence, the crosstalk between immune cells can be deemed a potential liver regeneration target.

## Conclusion

Our study found that Tet2 in macrophages negatively regulates liver regeneration. Tet2 interacted with Stat1 to inhibit the expressions of proinflammatory factors and suppress liver regeneration. Tet2 inhibitor BC339 attenuated the interaction of Stat1 and Tet2, enhanced Stat1 phosphorylation, and promoted hepatocyte proliferation. Our results suggest that targeting Tet2 in macrophages can be a therapeutic strategy for treating patients with HCC after liver resection, and the crosstalk between immune cells can be a potential target for liver regeneration.

### Supplementary Information

**Additional file 1: Supplemental Figure 1. **(A) Gene Ontology (GO) enrichment scatterplot of the top 20 pathways of macrophages at 0 and 24 h after PHx. (B) Mice mRNA levels of Tet2 in whole liver tissue 0, 24, and 48 h were detected using qPCR after PHx. The genes were normalized to GAPDH mRNA levels in each sample. (C) Kaplan–Meier analysis was used to determine the survival rate of mice after PHx with BC339 treatment. (A and B,*n*=3, C, *n*=10, *p* <0.05.).

**Additional file 2: Supplemental Figure 2.**

## Data Availability

The raw data generated in the study for macrophages have been deposited at Gene Expression Omnibus (GEO, GSE233516) and are publicly available as of the publication date. The data used in the Tet2 analysis in Fig. 3 was downloaded from the TCGA (https://portal.gdc.cancer.gov/), GEPIA (http://gepia.cancer-pku.cn/), and GEO (GSE158864, GSE124395) databases. The human liver cell atlas can be interactively explored at http://human-liver-cell-atlas.ie-freiburg.mpg.de/. The graphical abstract was depicted using Biorender online website (https://app.biorender.com/), and have got the publication licenses (Agreement number: IM260P8LES; TD260P8ELD).

## Abbreviations

ALB: Albumin
ALT: Liver transaminase-alanine aminotransferase
AST: Aspartate aminotransferase
BC339: Bobcat339
Cxcl10: C-X-C motif chemokine ligand 10
HCC: Hepatocellular Carcinoma
HDAC: Histone deacetylase
HGF: Hepatocyte growth factor
Hnf4α: Hepatocyte nuclear factor 4 alpha
Ifit1: Interferon-induced protein with tetratricopeptide repeats 1
IFN-γ: Interferon gamma
IL-6: Interleukin 6
Irf1: Interferon regulatory factor 1
ITA: Itaconic acid
ITGAM: Integrin alpha M
Jak1: Janus kinase 1
Jak2: Janus kinase 2
KCs: Kupffer cells
Ki67: Marker of proliferation Ki-67
Lgr5: Leucine-rich repeat-containing G protein-coupled receptor 5
LPCS: Liver progenitor cells
MD: Molecular dynamics
MFI: Mean fluorescence intensity
PD-1: Programmed cell death protein 1
