# Supplementary figures and images for: Ten-eleven translocation-2-mediated macrophage activation promotes liver regeneration
Source: Cell Commun Signal. 2024 Feb 2;22:95. doi: 10.1186/s12964-023-01407-7 (PMC10835877; doi:10.1186/s12964-023-01407-7)

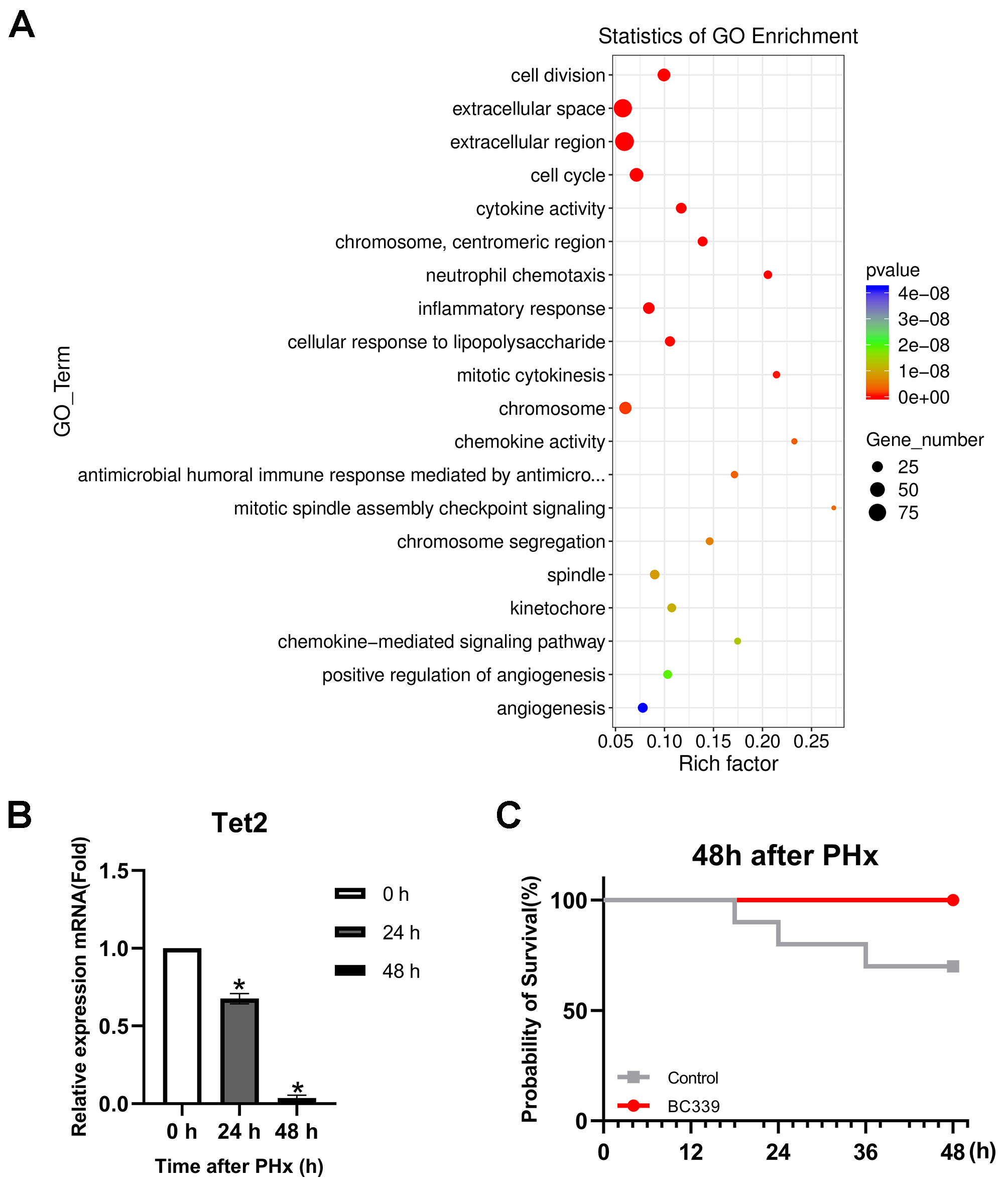

Supplement: Supplementary file 2 — Additional file 1: Supplemental Figure 1. (A) Gene Ontology (GO) enrichment scatterplot of the top 20 pathways of macrophages at 0 and 24 h after PHx. (B) Mice mRNA levels of Tet2 in whole liver tissue 0, 24, and 48 h were detected using qPCR after PHx. The genes were normalized to GAPDH mRNA levels in each sample. (C) Kaplan–Meier analysis was used to determine the survival rate of mice after PHx with BC339 treatment. (A and B,n=3, C, n=10, p <0.05.). [file 12964_2023_1407_MOESM1_ESM.jpg]

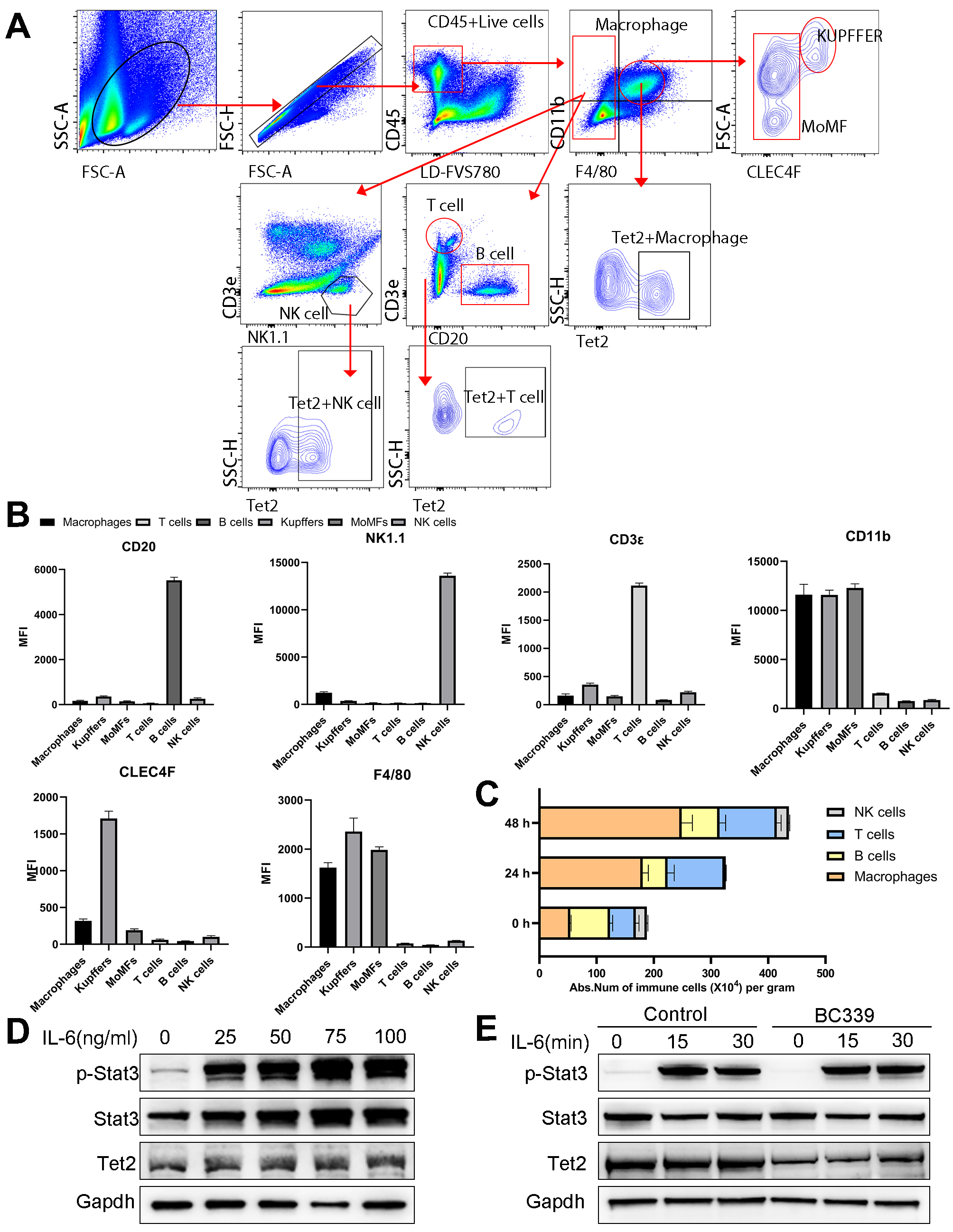

Supplement: Supplementary file 3 — Additional file 2: Supplemental Figure 2. [file 12964_2023_1407_MOESM2_ESM.jpg]
